# Tungsten disulfide atomic crystals with RONS scavenging and liver targeting capabilities for acetaminophen-induced acute liver injury therapy

**DOI:** 10.1186/s12951-025-03771-7

**Published:** 2025-10-11

**Authors:** Ziwen Xiao, Yu Liu, Zhenchao Tao, Yu Zhang, Qian Chen, Zhaohua Miao, Zhengbao Zha, Yan Ma, Hua Wang, Deyan Gong

**Affiliations:** 1 School of Food and Biological Engineering, Hefei University of Technology, Hefei, 230009 China; 2 Department of Radiation Oncology, Anhui Provincial Cancer Hospital, Hefei, 230031 China; 3 School of Biomedical Engineering, Anhui Medical University, Hefei, 230022 China; 4 Department of Oncology, The First Affiliated Hospital of Anhui Medical University, Hefei, 230032 China

**Keywords:** Acute liver injury, Pharmacology and toxicology, Two-dimensional nanomedicine, RONS scavenging, Tungsten disulfide

## Abstract

The ongoing progression of acetaminophen (APAP)-induced liver injury (AILI) resulting from excessive APAP intake can lead to acute liver failure (ALF). This process involves an imbalanced antioxidant system, upregulated inflammatory responses, and excessive accumulation of reactive oxygen and nitrogen species (RONS), which ultimately trigger apoptosis. In the present study, tannic acid-capped tungsten disulfide nanosheets (WS_2_@TA NSs), which are atomic crystals with a 2D structure, a high specific surface area, and abundant phenolic groups, were synthesized using a liquid-phase exfoliation method. WS_2_@TA NSs were investigated as highly effective anti-inflammatory nanomedicines for targeted enrichment therapy of AILI lesions. Enriched WS_2_@TA NSs in the liver were able to scavenge excess RONS, exhibiting potent antioxidant properties. Importantly, in addition of oxidative stress levels reducing, bioactive WS_2_@TA NSs, which are rich in variable valence states, were able to modulate multiple biosignaling pathways including Nrf2-Keap1, NF-κB, and apoptosis in advanced-stage AILI mice model, thereby enhancing liver tolerance to AILI. The present study reveals the clinical potential of WS_2_@TA NSs atomic crystals in targeted enrichment therapy for late-stage AILI lesions.

**Graphical abstract:**

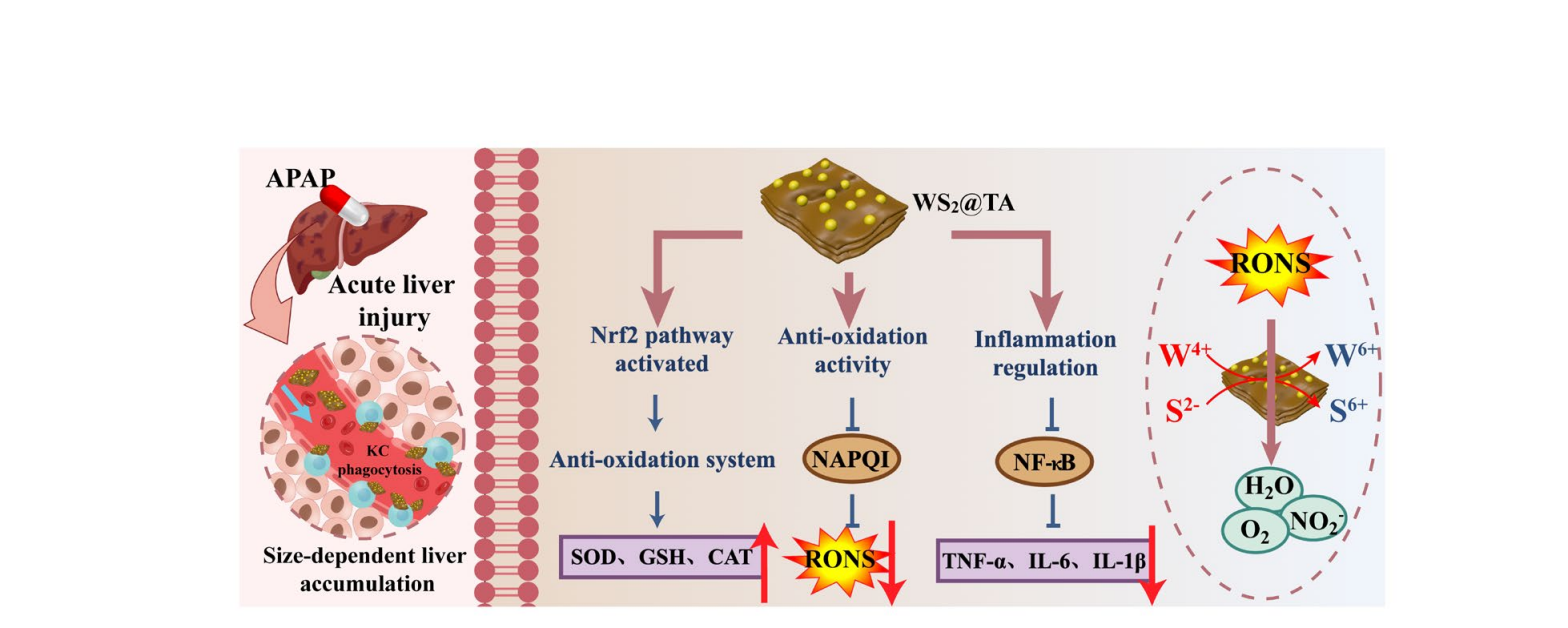

**Supplementary Information:**

The online version contains supplementary material available at 10.1186/s12951-025-03771-7.

## Introduction

Acetaminophen (APAP) is one of the most commonly used medications worldwide due to its significant analgesic and antipyretic effects [1]. APAP is used alone as well as extensively in compounded formulations and is often used in combination with over-the-counter or prescription opioids such as caffeine and antihistamines. Although APAP is safe and effective at recommended doses, overdose is its main cause of pharmacologic liver injury [2, 3]. As one of the classic cases in pharmacology and toxicology, APAP-induced liver injury (AILI) can further progress to acute liver failure (ALF), posing a major clinical challenge [4]. Currently, N-acetylcysteine (NAC) is the only medication approved for AILI, yet its efficacy in advanced AILI patients is uncertain [5]. The reason might be related to the fact that NAC primarily targets the oxidative stress damage induced by reactive metabolites that occur earliest after APAP ingestion rather than the sterile inflammation at the late stage of AILI [6, 7]. Therefore, the design and synthesis of novel medication for the treatment of AILI are crucial.

In the therapeutic dose range, only a small amount (5–10%) of APAP is metabolized through the cytochrome P450 (CYP, predominantly CYP2E1 isoforms) to produce the hepatotoxic active N-acetyl-p-benzoquinolinamine (NAPQI) [8]. Physiologically, NAPQI rapidly binds to the cysteine sulfhydryl group of glutathione (GSH) to form a NAPQI-GSH conjugate for detoxification [9]. However, APAP overconsumption will upset this balance. Overproduction of NAPQI drastically depletes hepatic GSH reserves, resulting in a massive coalescence of harmful reactive oxygen/nitrogen species (RONS) [10–12]. This process triggers mitochondrial oxidative stress, which ultimately leads to liver parenchymal cell injury. Notably, the pathological process of APAP-induced liver injury (AILI) is highly complex: in addition to oxidative stress, the mechanism involves the interaction of inflammatory cascade response, apoptosis, and other pathways [13]. Therefore, the development of efficient AILI therapeutic strategies requires comprehensive consideration of multiple factors: liver-targeted enrichment efficiency, RONS clearance capacity, and effective modulation of key signaling pathways.

Our group has strongly focused on the synthesis strategies and biomedical applications of nanomedicine in the diagnosis and treatment of digestive system diseases in recent years [14–18]. Moreover, our group has reported Mn_3_O_4_ nanozymes and manganese Prussian blue nanozymes with reactivity like catalase and superoxide dismutase (CAT and SOD), which effectively scavenge reactive oxygen species (ROS) and inhibit apoptosis through a multistep process of bioreactive modulation to counteract AILI, which are promising nanomedicines for the treatment of AILI [19, 20]. The tungsten-based nanomedicines have been demonstrated with promising biological activities for treating tumor or digestive system disease [21–23]. Two-dimensional nanomaterials are increasingly used in biomedical applications [24–27]. Among them, the emerging tungsten disulfide (WS_2_) nanomaterials show significant potential for antioxidant applications due to their unique physicochemical properties. WS_2_ nanosheets (WS_2_ NSs) have a high specific surface area, which can provide more active sites for RONS removal. Therefore, the development of a novel ROS-scavenging nanomedicine or natural antioxidant enzymes that can combine the advantages of both for more efficient and specific therapy has become an urgent technological problem in this field [28–33].

**Scheme 1:**
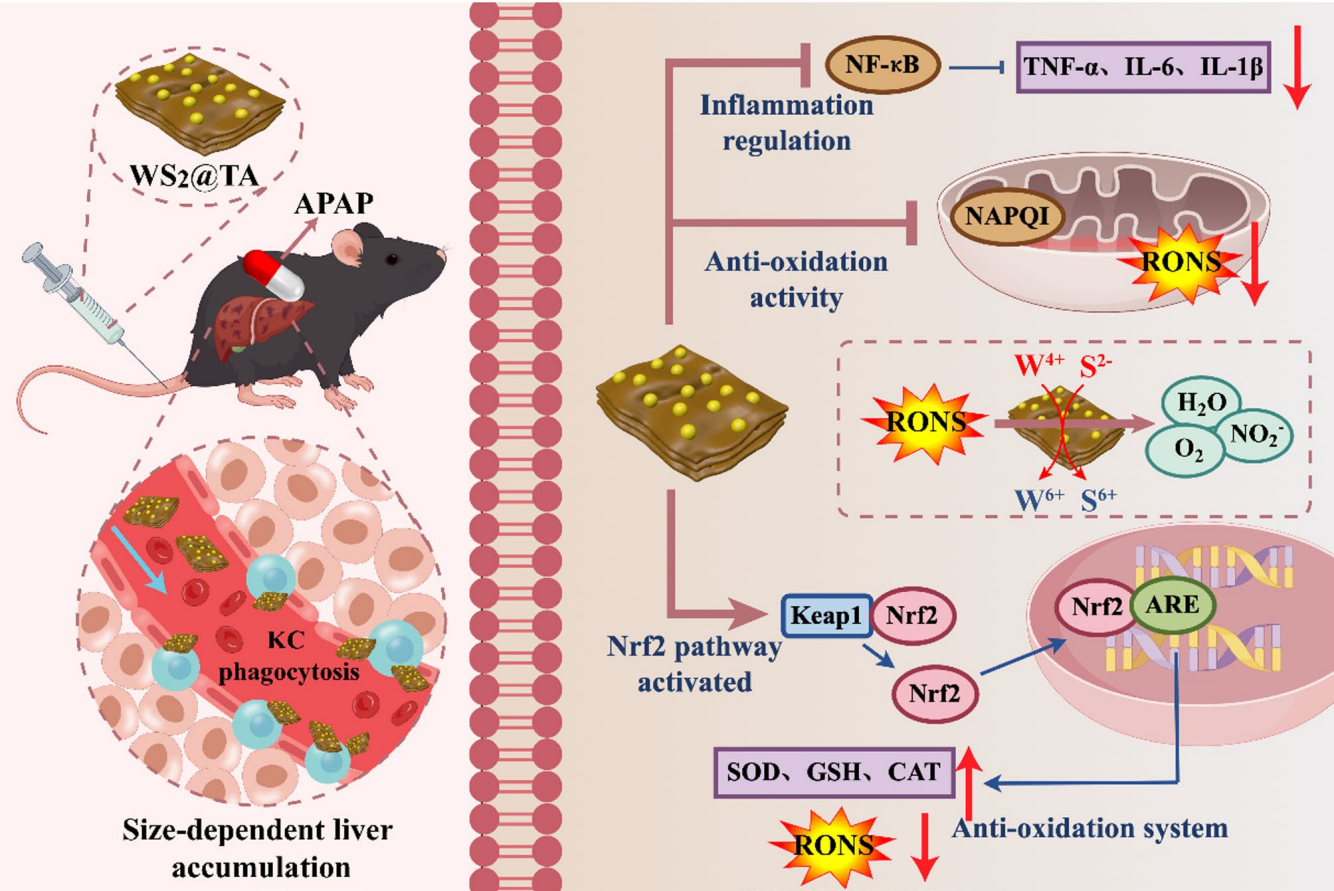
Schematic mechanism of WS_2_@TA NSs alleviating AILI mice models. WS_2_@TA NSs treatment attenuates AILI through several strategies. These strategies encompass the activation of the Nrf2 signaling pathway, the suppression of oxidative stress induced by mitochondrial dysfunction, and the regulation of inflammatory responses (The figure was drawn by Figdraw (www.figdraw.com))

A variety of natural products or their derivative nanomedicines have been applied in the treatment of liver injury [34]. Herein, we report tannic acid (TA)-coated WS_2_ nanosheets (WS_2_@TA NSs), which represent two-dimensional tungsten-based atomic crystal nanomaterials. TA is rich in phenolic hydroxyl and carboxyl groups, is not only a natural antioxidant, but also serves as a stabilizer to modify WS_2_ NSs. WS_2_@TA NSs can be used as high-performance antioxidant nanomedicines capable of eliminating RONS and down-regulating pro-inflammatory factors, thereby facilitating targeted enrichment therapy for AILI lesions (Scheme 1). The ultrathin WS_2_@TA NSs were synthesized using liquid-phase stripping method, which could eliminate various RONS through the valence transition from W^4+^ to W^6+^ on their surfaces and the synergistic oxidation reaction of S^2−^. Upon vein injection, the obtained WS_2_@TA NSs could be targeted and enriched at the site of the injured liver due to the size-mediated passive liver targeting, demonstrating good metabolism and biosafety. More importantly, WS_2_@TA NSs could activate Keap1-Nrf2 and inhibit the nuclear transcription factor (NF-κB) signaling pathway in the AILI mice model, thus achieving synergistic inflammation inhibition and effectively alleviating the advanced-stage AILI mice model. This study developed WS_2_@TA 2D atomic crystals with both excellent biocompatibility properties and antioxidant nanomedicine functionality, which can be precisely targeted for the treatment of advanced AILI lesions.

## Experimental section

### Materials

Tannic acid was purchased from Shanghai Aladdin Biochemical Technology Co., Ltd. Tungsten disulfide and Cy5.5 N-hydroxysuccinimide ester were purchased from Shanghai Macklin Biochemical Co., Ltd. ELISA enzyme-linked immunosorbent kit was purchased from MultiSciences (Lianke) Biotech Co., Ltd.

### Synthesis of WS_2_@TA

WS_2_@TA NSs were prepared by a liquid-phase stripping strategy. The pristine WS_2_ powder (200 mg) was immersed in liquid nitrogen for 1 h to condition its mechanical characteristics in an ultra-low temperature. Next, the cryoprepared WS_2_ powder was immediately transferred into an aqueous tannic acid solution (20 mg/mL, 30 mL) for probe sonication (480 W) for 5 h. After that, the dispersion was centrifuged at 3000 rpm for 20 min to get rid of the WS_2_ powder. The resulting overlying liquid was further centrifuged (12000 rpm, 20 min) to collect the precipitate and washed three times with deionized water to obtain WS_2_@TA NSs. The obtained WS_2_@TA NSs dispersion was kept at 4 °C.

### Characterization

The morphology and lattice of WS_2_ NSs and WS_2_@TA NSs were researched by field emission transmission electron microscopy. The hydrodynamic diameters and zeta potentials of WS_2_ NSs and WS_2_@TA NSs were analyzed by particle size potentiometry. The elemental chemical states of WS_2_@TA NSs were analyzed by X-ray photoelectron spectroscopy. The crystal structure of WS_2_@TA NSs was analyzed by rotary-target X-ray diffractometry.

### RONS scavenging ability test

To assay the ability of WS_2_@TA NSs to scavenge ABTS radicals, a mixture containing potassium persulfate (1.6 mM) and ABTS (2.4 mM) stock solution was incubated for 12 h. 40 µL of WS_2_ NSs and WS_2_@TA NSs solutions at different concentrations (0, 9.4, 18.8, 37.5, and 75 µg/mL) were combined with 160 µL ABTS solution and incubated for 10 min. Then 734 nm absorbance of the resulting mixture were recorded. DPPH scavenging capacity was measured as described previously. For ·OH scavenging, FeSO_4_ (1 mM) was reacted with H_2_O_2_ (5 mM) for 15 min to obtain ·OH. Subsequently, varying concentrations of WS_2_ NSs and WS_2_@TA NSs were added, and the reaction was conducted at 37 °C for 1 h. Then, salicylic acid solution (1.8 mM) was added. After incubation for 15 min, the absorbance of 510 nm were quantified using a spectrophotometer [35].

### Cell toxicity assessment

In 96-well plates, Alpha Mouse Liver 12 (AML12) cells were incubated for 12 h. WS_2_@TA NSs were added and the cells were incubated for an additional 24 h. 100 µL of MTT solution (0.5 mg/mL) was added and maintained for 3–4 h. The supernatant was carefully removed and 150 µL of dimethyl sulfoxide (DMSO) was added. The cells were then incubated at 37 °C for 10 min. Finally, the 570 nm absorbance was measured to determine cell viability.

### Cellular anti-inflammatory and antioxidant efficacy testing

To test RONS elimination efficacy of WS_2_@TA NSs, AML 12 cells were incubated for a duration of 2 h with WS_2_@TA NSs (200 µg/mL) or NAC (4 µg/mL), followed by exposure to H_2_O_2_ (1 mM) for 4 h. Subsequently, 10 µM DCFH-DA was added, and the cells were incubated for 30 min. All procedures were conducted in a dark environment. Afterward, the cells were purged twice with PBS, RONS levels were evaluated by fluorescence microscopy. The protective effect of WS_2_@TA NSs against H_2_O_2_-induced oxidative damage was evaluated by fluorescence live/dead assay. AML12 cells treated with 3 mM H_2_O_2_ were incubated with or without WS_2_ NSs and WS_2_@TA NSs. Then, 500 µL of mixed dye (calcein-AM/PI, 15 µL:10 µL) was added and incubated for 30 min. The dye was aspirated, and 500µL of PBS was added to each well to obtain fluorescence images under a fluorescence microscope. The anti-inflammatory effects of WS_2_@TA NSs at the cellular level were assessed by incubating LPS (1 µg/mL) pretreated RAW 264.7 cells with WS_2_ NSs (100 µg/mL) and WS_2_@TA NSs (100 µg/mL) for 12 h, respectively. Pro-inflammatory cytokine levels were detected using the corresponding ELISA kits.

### In vivo biocompatibility assessment

C57BL/6 mice (20–22 g, male) were acquired from Henan Sikebas Biotechnology Co. Ltd. Mice in the WS_2_@TA group were intravenously administered 10 mg/kg WS_2_@TA NSs, and mice in the control group were administered PBS on day 0 (*n* = 3). Mice body weights were monitored over a period of 28 consecutive days. Hearts, livers, spleens, lungs, kidneys and blood were collected on days 3, 7, and 28, respectively. 2 h after blood collection, the supernatant was subjected to centrifugation at 3000 rpm for 20 min for serum collection. The serum levels of alanine aminotransferase (ALT), aspartate aminotransferase (AST), urea nitrogen (UREA), uric acid (UA), and total protein (TP) were quantified using an automatic biochemical analyzer; meanwhile, hemoglobin (HGB), red blood cells (RBC), and white blood cells (WBC) levels were assessed utilizing an automatic blood cell analyzer. H&E staining was also performed on the main organs of mice.

### In vivo targeting and distribution experiments

Cy5.5-labeled WS_2_@TA NSs were prepared by mixing 0.01 mg of Cy5.5 dye with 1 mg of WS_2_@TA NSs for 12 h, followed by centrifugation (12,000 rpm, 20 min), then the precipitates were collected and washed twice with deionized water. C57BL/6 mice underwent a fasting period of 14 h and were then administered an intraperitoneal injection of APAP (300 mg/kg) to establish AILI mice model, and healthy mice served as controls. Then, mice were injected with Cy5.5-labeled WS_2_@TA NSs (*n* = 3, 2.5 mg/kg). Major organs were collected at 2, 6, 12, and 24 h after the administration of cytotoxicity, and dark conditions were used to observe fluorescent signals or ICP-MS measurements.

### AILI mice models

The advanced-stage AILI mice models were given an intraperitoneal injection of APAP (300 mg/kg) after 14 h of fasting. Subsequently, mice were allowed to eat and drink freely. Typical symptoms of AILI, such as loss of appetite and reduced activity, could be observed within a few hours after injection.

### In vivo delayed therapeutic effect of WS_2_@TA NSs on AILI mice

First, 300 mg/kg APAP was injected transperitoneally to construct the advanced-stage AILI mice model, and NAC (intravenous injection, 300 mg/kg), WS_2_ NSs (intravenous injection, 2.5 mg/kg), and WS_2_@TA NSs (intravenous injection, 2.5 mg/kg) were administered 3 h later. After 24 h of drug administration, mice were euthanized and blood was collected for transaminases (ALT, AST) detection, while mice livers were collected for H&E staining.

### Survival curves of AILI mice

Mice that underwent a fasting period of 14 h received intraperitoneal injections of APAP (600 mg/kg) for 3 h (to establish the APAP lethal dose mice model), followed by administration of PBS, NAC (300 mg/kg) and WS_2_@TA NSs (2.5 mg/kg), respectively. The number of surviving mice were counted every 8 h for the following 5 days, and survival curves were plotted.

### Antioxidant capacity of WS_2_@TA NSs

The antioxidant capacity of liver tissue was assayed by CAT, SOD and GSH assay kits, whereas the lipid peroxidation response of the liver was assessed using a malondialdehyde (MDA) assay kit.

### Protein extraction and Western blotting (WB)

Total proteins were isolated from liver tissues utilizing an animal whole protein extraction kit. The extracted proteins were mixed with the loading buffer and subjected to electrophoresis on 10% sodium dodecyl sulfate polyacrylamide gel (SDS-PAGE), the proteins were then transferred to polyvinylidene difluoride (PVDF) membrane and blocked with 5% skimmed milk powder solution for a duration of 2 h. The PVDF membranes were incubated with the primary antibody overnight at 4 °C and then with the corresponding secondary antibody for 2 h at room temperature. Anti-Nrf2, anti-Keap1, anti-NF-κB, anti-p65, and anti-IκBα were purchased from CST, and anti-β-actin was purchased from ABclonal.

### Statistical analysis

Data were statistically assessed using one-way ANOVA. Statistically significant differences were established with the following criteria: n.s. (no significance), *p < 0.05, **p < 0.01, and ***p < 0.001.

## Results and discussion

### WS_2_@TA NSs Preparation and characterization

**Fig. 1:**
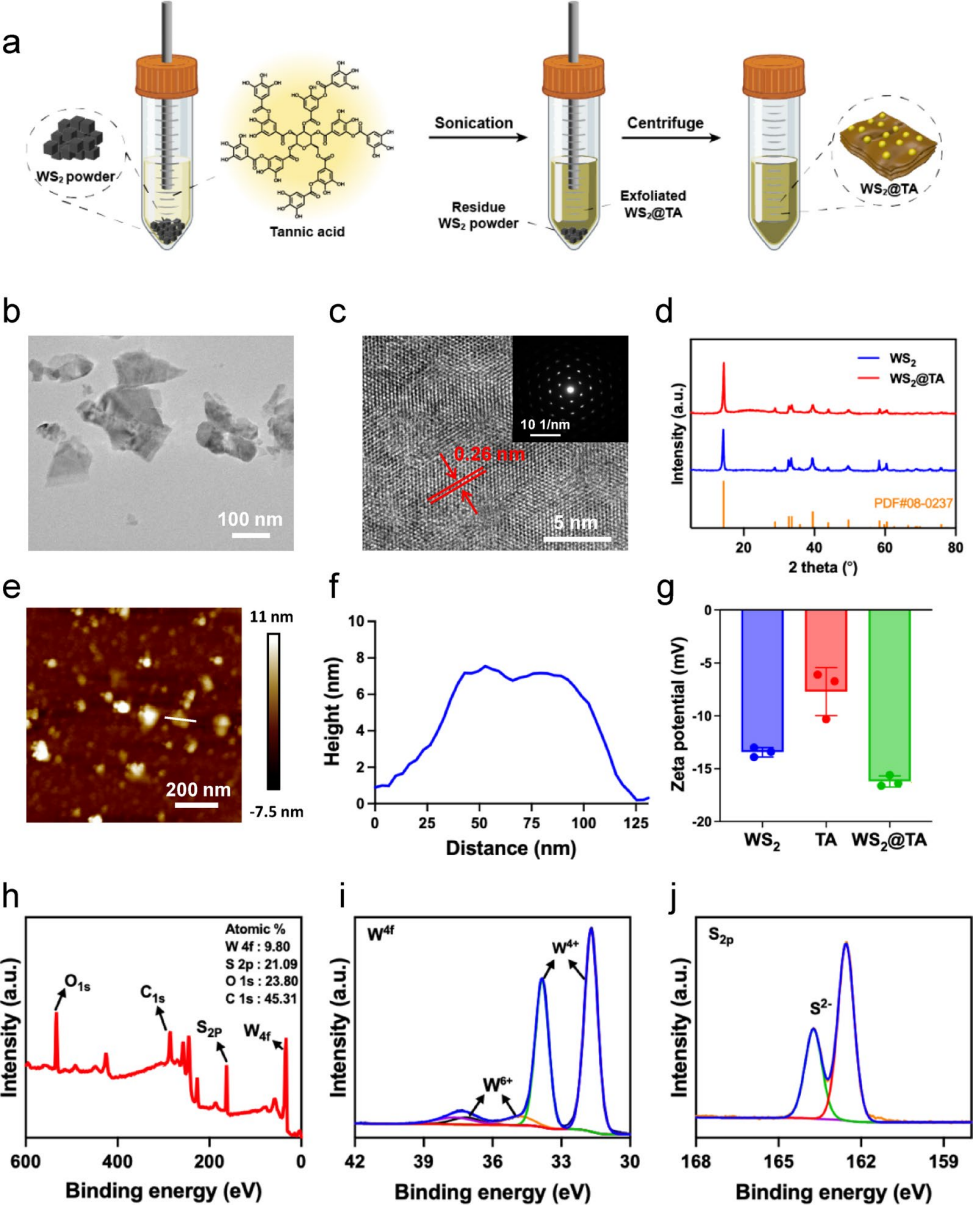
Synthesis and characterization. (**a**) Schematic diagram of the liquid-phase stripping process of WS_2_@TA NSs. (**b**) TEM image of WS_2_@TA NSs. (**c**) HRTEM image of WS_2_ NSs. The corresponding SAED diffraction patterns are presented in the insets. (**d**) XRD of WS_2_@TA NSs and WS_2_ NSs. (**e**) AFM image, along with (**f**) thickness profiles of WS_2_@TA NSs. (**g**) ζ potential of WS_2_@TA NSs and WS_2_ NSs. (**h**) XPS spectra of WS_2_@TA NSs. (**i**) Core XPS spectra of W element. (**j**) Core XPS spectra of S element

WS_2_@TA NSs were synthesized through liquid phase stripping method, in which cryoprocessed WS_2_ powder and tannic acid were dispersed in water and sonicated using probe sonication for 5 h. The cryoprocessed WS_2_ powder and tannic acid were then removed by centrifugation, and the cryoprocessed WS_2_@TA NSs were finally prepared. Unstripped WS_2_ powder and unmodified tannic acid were removed by centrifugation, thus obtaining the purified WS_2_@TA NSs (Fig. 1a). The WS_2_ NSs and WS_2_@TA NSs were morphologically characterized using transmission electron microscopy (TEM) and atomic force microscopy (AFM). The representative TEM images showed that the obtained WS_2_ NSs and WS_2_@TA NSs had transverse dimensions of 100–200 nm (Fig. 1b, Fig. S1a). High-resolution transmission electron microscopy (HRTEM) images demonstrated that the lattice spacing of WS_2_@TA NSs was 0.26 nm, which corresponded with the {101} crystallographic facets of the element (Fig. 1c). In addition, selective electron diffraction (SAED) patterns further confirmed the single-crystal nature of the WS_2_@TA NSs (inset in Fig. 1c). X-ray diffraction (XRD) patterns exhibited a strong correlation with the reference standard card (JCPDS PDF card No. 08–0237), suggesting that the crystalline structure is still maintained after exfoliation (Fig. 1d). The AFM images indicated that the thickness of the WS_2_ NSs was about 5 nm (Fig. S1b, c), while the thickness of WS_2_@TA NSs was slightly higher than that of WS_2_ NSs, which was attributed to the surface-coated TA (Fig. 1e, f). Dynamic light scattering (DLS) measurements indicated that the hydrated particle size of WS_2_ NSs was approximately 200 nm, with a PDI of 0.305. While the hydrated particle size of WS_2_@TA NSs was about 140 nm, corresponding to a PDI of 0.207 (Fig. S2). Regarding size stability assessment, particle size stability measurements of WS_2_@TA NSs and WS_2_ NSs in water, serum, and physiological saline were assessed. WS_2_ NSs exhibits poor size stability in physiological saline, prone to aggregation and sedimentation (Fig. S3). One of the functions of biomolecule TA is to act as a stabilizer to modify WS_2_ NSs. Particle size of WS_2_@TA remained virtually unchanged over three days in different media, demonstrated significantly enhanced size stability via TA modification impact. UV-vis spectroscopy observed that the 200–250 nm absorbance of WS_2_@TA NSs was higher than that of WS_2_ NSs (Fig. S4), which indicated that TA had been successfully modified onto WS_2_ NSs. TA exhibited a high affinity for the nanosheets during the stripping process, significantly improving their stability in aqueous environments. Additionally, the presence of numerous carboxyl and hydroxyl groups in TA further consolidated this stability by decreasing the potential of WS_2_ from − 13.43 ± 0.45 mV to −16.2 ± 0.53 mV after capping with TA (Fig. 1g). Fourier transform infrared (FTIR) spectra showed two peaks at 3368 cm^−1^ and 760 cm^−1^ for WS_2_@TA, which corresponded to OH and C-O-C in TA (Fig. S5). Regarding the quantitative analysis of TA grafting efficiency, calculation from TGA analysis results of WS_2_@TA NSs showed the load rate of TA in WS_2_@TA NSs is 6.9% (Fig. S6). X-ray photoelectron spectroscopy (XPS) of WS_2_@TA NSs showed five major peaks (Fig. 1h), which were attributed to W4f (9.8 at%), S2p (21.09 at%), O1s (23.80 at%), and C1s (45.31 at%). High-resolution spectral analysis revealed the presence of four carbon types and two oxygen types in the WS_2_@TA NSs (Fig. S7), indicating the presence of O-C = O, C-C, and C-O-C groups in the WS_2_@TA NSs, which together confirmed the successful modification of TA. High-resolution element spectral analysis displayed that tungsten has a predominant valence state of + 4 and sulfur has a predominant valence state of −2. (Fig. 1i, j), indicating the potential antioxidant capacity of the prepared WS_2_@TA NSs.

### RONS scavenging and Proinflammatory cytokine downregulation in vitro

**Fig. 2:**
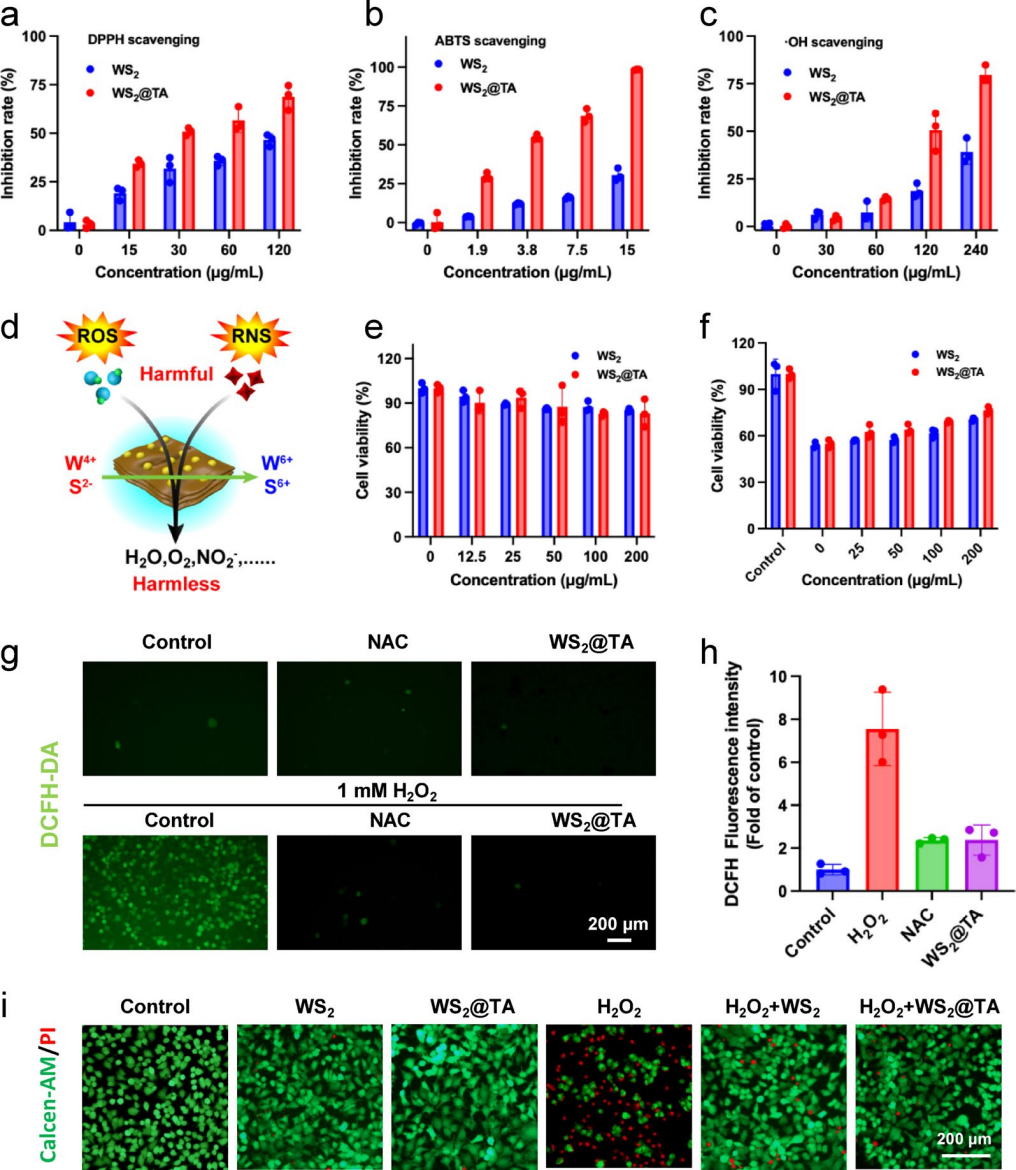
RONS scavenging activities. (**a**) DPPH, (**b**) ABTS, and (**c**) •OH scavenging efficiencies. (**d**) Illustration of the RONS scavenging mechanism of WS_2_@TA NSs. (**e**) Cell viability of AML12 cells treated with WS_2_ NSs or WS_2_@TA NSs of different concentrations (*n* = 3). (**f**) Cell viability of AML12 cells treated with APAP, followed by treatment with WS_2_ NSs or WS_2_@TA NSs. (**g**) WS_2_@TA NSs and NAC in AML12 cells treated with 1 mM H_2_O_2_, as measured by DCFH-DA staining. (**h**) The corresponding mean fluorescence intensity of (**g**). (**i**) Fluorescence images of 3 mM H_2_O_2_-treated AML12 cells after various treatments

Excess RONS in the inflammatory zone are closely associated with the progression of AILI. Due to the − 2 valence of sulfur and + 4 valence of tungsten in WS_2_@TA NSs, they are potentially reductive and can achieve effective scavenging of RONS. Therefore, the scavenging ability of WS_2_@TA NSs for a variety of RONS was first investigated. As shown in Fig. 2a and b, WS_2_ NSs and WS_2_@TA NSs could effectively scavenge ABTS and DPPH. WS_2_@TA NSs exhibited excellent RONS scavenging efficiencies, with DPPH scavenging efficiency 1.48 times higher than that of WS_2_ NSs at 120 µg/mL, and the scavenging efficiency of DPPH was 1.48 times higher than that of WS_2_ NSs at a concentration of 15 µg/mL. ABTS scavenging efficiency was 3.22-fold that of WS_2_ NSs. The DPPH scavenging kinetic curves of WS2@TA and WS_2_ at a concentration of 150 µg/mL also demonstrated that TA-modified WS_2_@TA possesses higher and faster scavenging capacity (Fig. S8). In addition to RNS, the ROS scavenging ability was also evaluated. WS_2_ NSs and WS_2_@TA NSs could effectively eliminate hydroxyl radical (·OH) and H_2_O_2_ at 240 µg/mL (Fig. 2c, S9), The scavenging efficiency of WS_2_@TA NSs for ·OH was 2.04-fold than that of WS_2_ NSs, while their H_2_O_2_ scavenging efficiency was 1.7-fold than that of WS_2_ NSs. These results clearly indicated that WS_2_@TA NSs exhibited superior RONS scavenging efficacy compared with WS_2_ NSs, which may be closely related to the phenolic moieties enriched on the surface of WS_2_@TA NSs. In addition, XPS spectra of WS_2_@TA NSs after 1 h incubation with H_2_O_2_ (8 mM) revealed that the W^4+^/S^2−^ peak rapidly decreased while new peaks of W^6+^/S^6+^ emerged after H_2_O_2_ treatment, indicating the change of W^4+^/S^2−^ to W^6+^/S^6+^ during the process of H_2_O_2_ scavenging (Fig. 2d and Fig. S10). XPS results demonstrate that RONS scavenging capacity of WS_2_@TA NSs was mainly attributed to the S^2−^/S^6+^ and W^4+^/W^6+^ valence shifts.

The favorable RONS scavenging ability of WS_2_@TA NSs led to further validation of its safety and efficacy at the cellular level. First, WS_2_@TA NSs did not show significant cytotoxicity after being incubated with various concentrations of AML 12 cells and Raw 264.7 cells for 24 h, suggesting that WS_2_@TA NSs have the expected biocompatibility (Fig. 2e, S11). In addition, the protective effect of WS_2_@TA NSs on APAP-stimulated AML 12 cells were validated using the MTT assay. The results indicated that WS_2_@TA NSs enhanced cell viability by 1.4-fold at 200 µg/mL (Fig. 2f).

The accumulation of ROS can cause cytotoxicity, including inflammatory response, oxidative stress, apoptosis, and autophagy. The ability of WS_2_@TA NSs to efficiently scavenge ROS at the cellular level was evaluated by modeling the oxidative stress environment in H_2_O_2_-treated AML12 cells (Fig. 2g, h). Intracellular ROS were stained with DCFH-DA fluorescent dye. Green fluorescence was negligible in the 1 mM H_2_O_2_ + WS_2_@TA NSs-treated group, which can be ascribed to the better ROS scavenging capacity of WS_2_@TA NSs. The cytoprotective ability of WS_2_@TA NSs was then assessed by live/dead fluorescence staining assay (Fig. 2i). Compared with the large number of AML12 cell deaths in the 3 mM H_2_O_2_-treated group, there were few dead cells in the WS_2_@TA NSs (200 µg/mL) or 3 mM H_2_O_2_ + 200 µg/mL WS_2_@TA NSs-treated groups.

LPS treated RAW 264.7 cells produce a large number of pro-inflammatory mediators, specifically IL-1β, TNF-α, and IL-6 [36]. Therefore, the in vitro anti-inflammatory effects of WS_2_@TA NSs were assessed using LPS as an exogenous stimulus to induce macrophage differentiation. WS_2_@TA NSs significantly down-regulated various pro-inflammatory cytokines induced by LPS (Fig. S12 a-c). This suggests that WS_2_@TA NSs can maintain cellular oxidative stress homeostasis by effectively scavenging RONS and down-regulating pro-inflammatory cytokines, resulting in a favorable anti-inflammatory effect in vitro.

### Cellular internalization and in vivo distribution of WS_2_@TA NSs

**Fig. 3:**
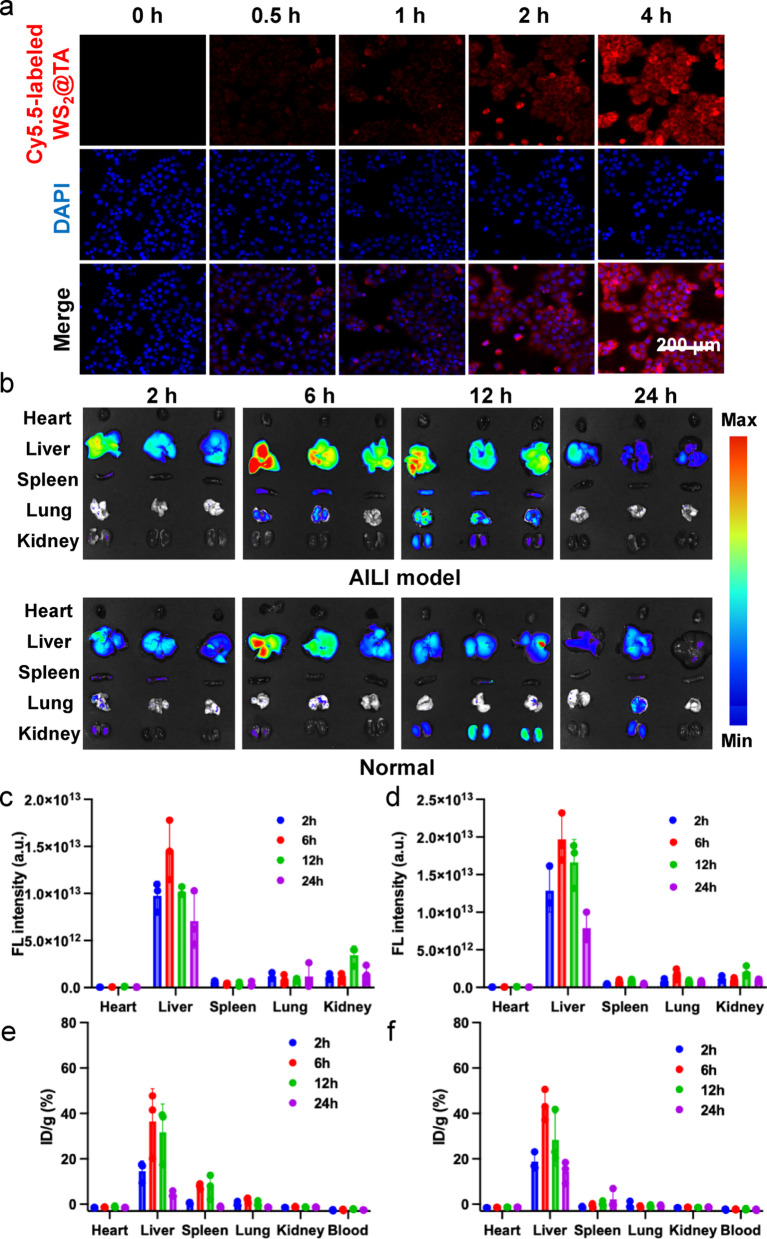
(**a**) Fluorescence images of AML12 cells treated with Cy5.5-labeled WS_2_@TA for different times. (**b**) At 2, 6, 12, and 24 h, Cy5.5-labeled WS_2_@TA in vitro distribution fluorescence images of major organs in AILI mice model and normal mice. (**c**) Quantitative analysis of Cy5.5 labeled WS_2_@TA fluorescence intensity in normal mice organs and (**d**) AILI mice organs (*n* = 3). Distribution of W element ICP-MS measurements in organs of (**e**) healthy mice and (**f**) AILI mice after intravenous injection of WS2@TA (*n* = 3)

To verify the in vitro uptake of WS_2_@TA NSs, Cy5.5-labeled WS_2_@TA NSs were incubated with AML12 cells. The experimental results showed that Cy5.5-labeled WS_2_@TA had excellent in vitro cellular uptake after 4 h of incubation (Fig. 3a, S13), a result that strongly confirmed the efficient internalization of WS_2_@TA NSs in AML12 cells. Limulus amebocyte lysate (LAL) detection of endotoxin showed that the endotoxin content in a 0.5 mg/mL WS_2_@TA NSs solution was 0.02 EU/mL, falls far below specific safety standards, confirms that WS_2_@TA NSs is safe for intravenous administration (Fig. S14). The hemolysis assay showed that no significant hemolysis was observed after incubation of blood with various concentrations of WS_2_@TA NSs (Fig. S15). At a concentration of 200 µg/mL, the hemolysis rate of WS_2_@TA remained below 10%, indicating its good blood biocompatibility. Then, the in vivo biodistribution of WS_2_@TA NSs was investigated after being intravenously injected into AILI mice and healthy mice, respectively. The results showed that a large amount of Cy5.5-labeled WS_2_@TA reached the major organs via blood circulation. The liver exhibited the highest concentration of WS_2_@TA NSs, while there was a significant decreasing trend of the residual amount in the major organs after 24 h (Fig. 3b-d). Meanwhile, the distribution of WS_2_@TA NSs in the liver of AILI mice were larger than that of normal mice, which was attributed to the TA functionalization on the surface of the nanosheets. The experimental results showed that WS_2_@TA NSs exhibited significant targeted enrichment at the site of hepatic injury and had a longer liver retention time. The concentration of WS_2_@TA NSs in the kidneys of AILI mice and healthy mice reached a peak at 12 h, and then decreased, which indicated that some of the WS_2_@TA NSs were excreted through urine, and that the metabolism of WS_2_@TA NSs was well behaved and did not remain in the body for a long period of time. According to existing research [37], nanoparticles with a diameter of approximately 100–150 nm can traverse hepatic sinusoids and accumulate within hepatocytes. WS_2_@TA NSs fall precisely within this size range, suggesting that size effects is the primary mechanism for WS_2_@TA efficient enrichment in the liver. To validate this hypothesis, ICP-MS measurements of W residue in major organs were performed 24 h after injection of WS_2_ and WS_2_@TA (intravenous injection, 2.5 mg/kg) in normal mice and AILI model mice. Results showed both nanomaterials significantly enriched in the liver (Fig. 3e-f). Comparative analysis revealed that TA modification had no significant effect on the targeted enrichment of WS_2_ NSs in the liver of normal mice and AILI model mice (Fig. S16). This finding further supports the dominant role of the size effect in liver targeting. Additionally, 12 h post-injection, tungsten residues in mice began to decrease significantly, indicating that WS_2_@TA NSs exhibit favorable metabolic clearance properties.

### Biocompatibility of WS_2_@TA NSs

**Fig. 4:**
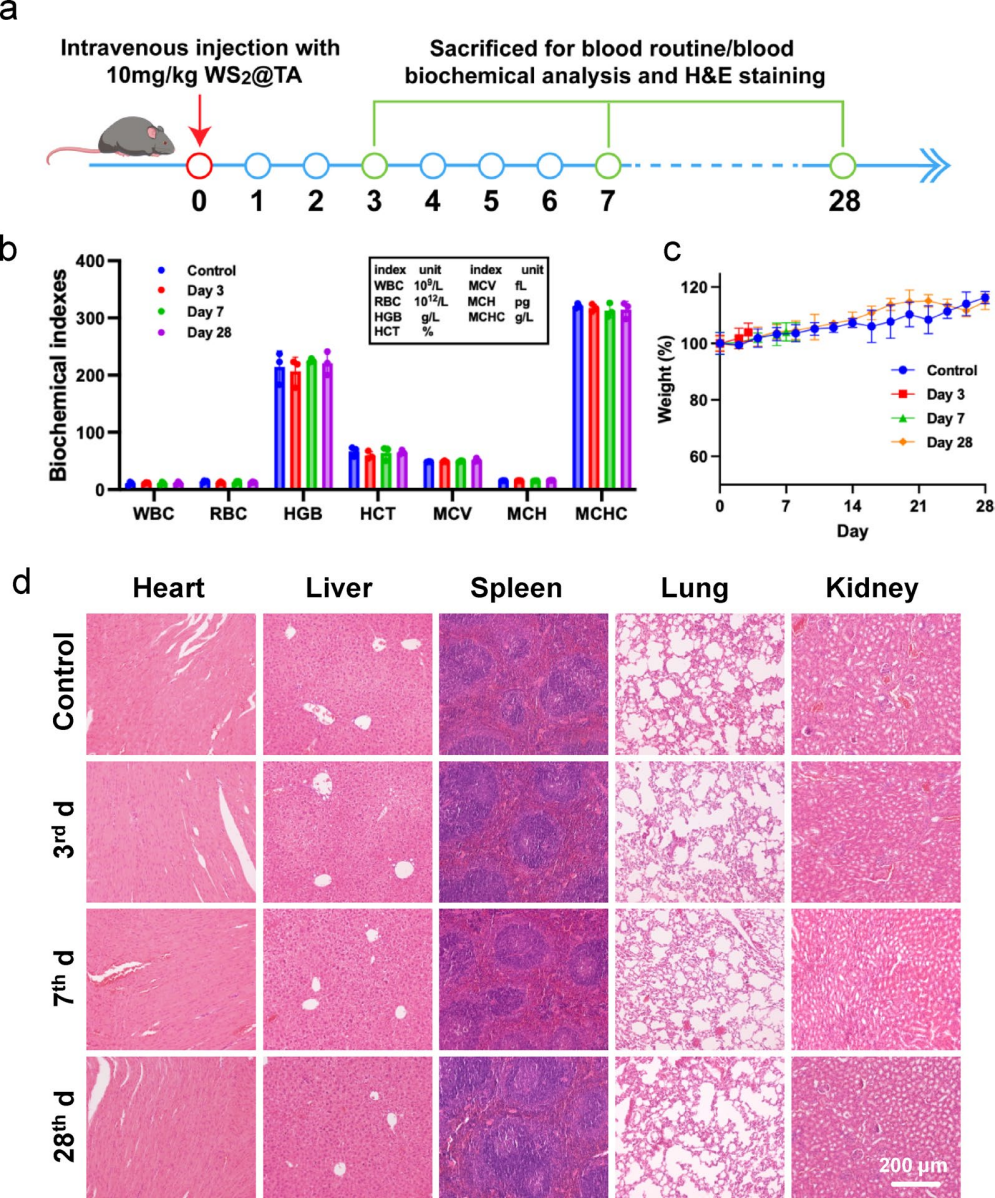
Biocompatibility and biodistribution of WS_2_@TA NSs. (**a**) Schematic diagram of the in vivo biosafety experiment program. (**b**) Blood tests. (**c**) Body weights change of different mice group were monitored over different period. (**d**) Observations on H&E staining of organs in healthy mice on days of 3, 7, and 28 after injection of WS_2_@TA NSs

The biocompatibility of WS_2_@TA NSs in vivo was evaluated before its application to AILI treatment. After the injection of WS_2_@TA NSs at a dosage of 10 mg/kg through the tail vein on day 0, the body weights of the mice were monitored daily. Major organs and blood were collected from the mice by euthansia on days 3, 7, and 28, for H&E staining and analysis of blood indices, respectively (Fig. 4a). No statistically significant differences were noted in blood parameters, including red blood cells (RBC), white blood cells (WBC), mean corpuscular hematocrit (HCT), hemoglobin (HGB), mean corpuscular hemoglobin volume (MCH), mean corpuscular volume (MCV), and mean corpuscular hemoglobin concentration (MCHC) (Fig. 4b). In addition, typical liver and kidney function indices, including alanine aminotransferase (ALT), aspartate aminotransferase (AST), urea (UREA), uric acid (UA), and total protein (TP), did not show significant changes in the different treatment groups, demonstrating that WS_2_@TA NSs do not have significant toxic effects on the liver and kidneys (Fig. S17). Furthermore, there were no significant difference in body weights gain over 28 days in mice administered with WS_2_@TA NSs compared to the control mice (Fig. 4c). Meanwhile, H&E staining showed no obvious pathological changes or inflammatory lesions in major organs tissues (Fig. 4d). The experimental results showed that WS_2_@TA NSs had good biocompatibility, which ensured the safety of WS_2_@TA NSs for AILI treatment.

### Delayed therapeutic effect of WS_2_@TA on AILI

**Fig. 5:**
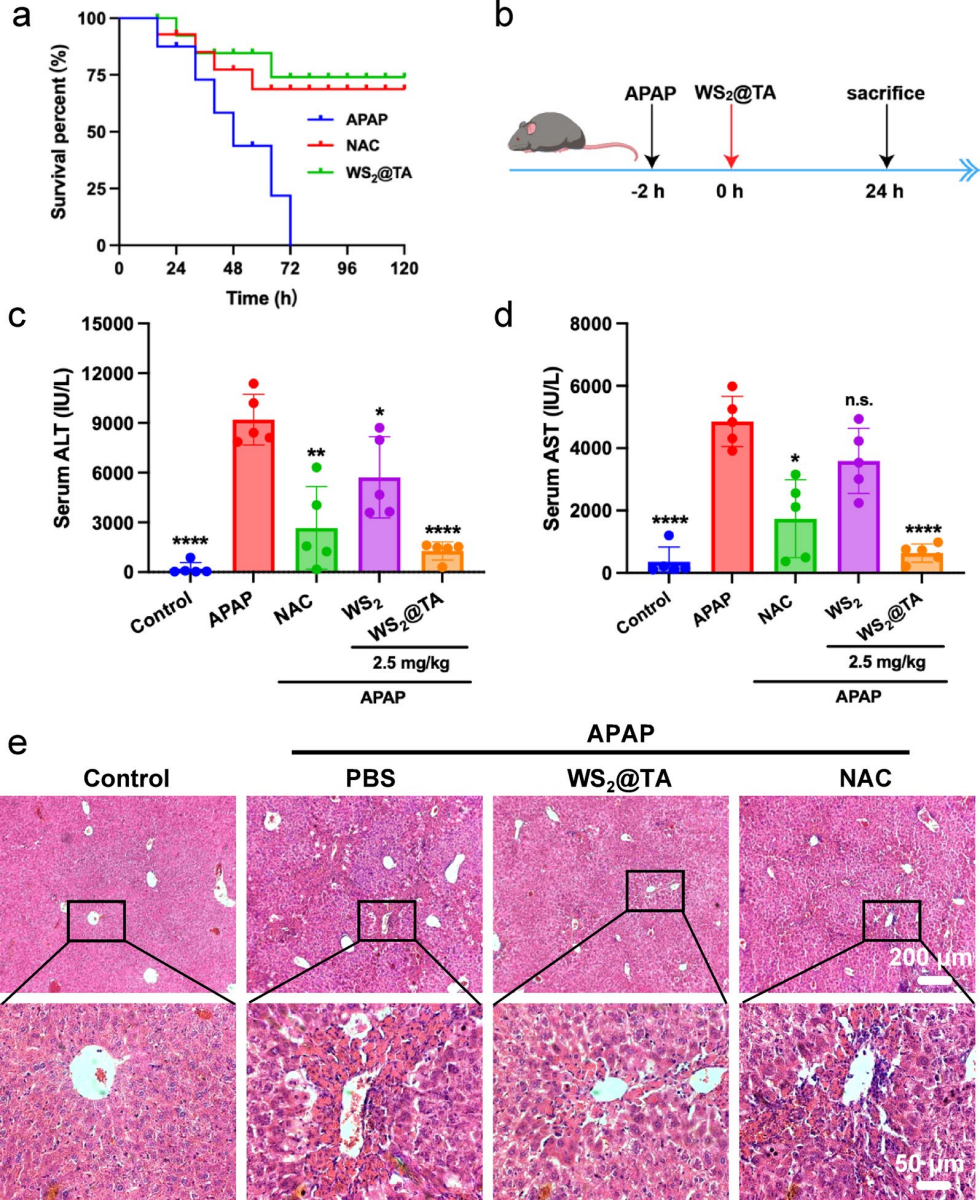
Therapeutic effect of WS_2_@TA NSs against AILI. (**a**) Survival curves of AILI mice in PBS, NAC and WS_2_@TA NSs treated groups 3 h after APAP (600 mg/kg) administration (*n* = 6). (**b**) Schematic diagram of the protocol for WS_2_@TA treatment of advanced-stage AILI mice. (**c**) Serum AST and (**d**) ALT levels in different mice group (*n* = 5). (**e**) H&E-stained liver tissue observations

The therapeutic effects of WS_2_@TA NSs on AILI mice were evaluated because of their excellent RONS scavenging and excellent biocompatibility properties. C57 mice were fasted for 14 h. APAP lethal dose mice model were induced by intraperitoneal injection of high-dose APAP (600 mg/kg), which was administered intravenously in the tail vein 3 h after fasting. The survival rates of the mice were documented at 8 h intervals, and 120 h continuously recording for the survival curve. As can be seen in Fig. 5a, the survival rate of mice treated with 2.5 mg/kg WS_2_@TA was significantly higher than that of the control group treated with APAP. In addition, the survival rate of this treatment group was also superior to that of mice treated with the clinical drug NAC. Next, the therapeutic effect of WS_2_@TA NSs on the model of AILI mice were evaluated. C57 mice were randomly assigned to 5groups: the PBS group, the APAP + PBS group, the APAP + NAC (300 mg/kg) group, the APAP + WS_2_ NSs (2.5 mg/kg) group, and the group of APAP + WS_2_@TA NSs (2.5 mg/kg). The mice underwent a 14-hour fasting period before APAP (300 mg/kg) administered via intraperitoneal injection to establish the AILI mice model for treatment testing. 3 h later, the mice received different treatment, and 24 h later, they were euthanized (Fig. 5b), and serum ALT and AST were used as the basic indexes for assessing liver injury. As expected after a 24 h period, the levels of AST and ALT were significantly elevated in mice subjected to APAP overdose, thereby confirming the successful AILI mice model establishment. In addition, AILI mice treated with WS_2_@TA NSs exhibited significantly lower serum ALT and AST levels, where the low dose of WS_2_@TA NSs (2.5 mg/kg) was superior to NAC (300 mg/kg) in the treatment of AILI (Fig. 5c, d). This may be due to the enrichment of WS_2_@TA NSs in the liver site and its longer in vivo retention time. Histological observations of liver tissues showed significant improvement in liver-related pathologic lesions after treatment with WS_2_@TA NSs, which significantly reduced hepatic sinusoidal dilation, infiltration of inflammatory cells, and hepatocyte death induced by APAP administration (Fig. 5e), confirming the superior therapeutic efficacy of WS_2_@TA NSs in the treatment of AILI.

### The mechanism of WS_2_@TA NSs in AILI mice models

**Fig. 6:**
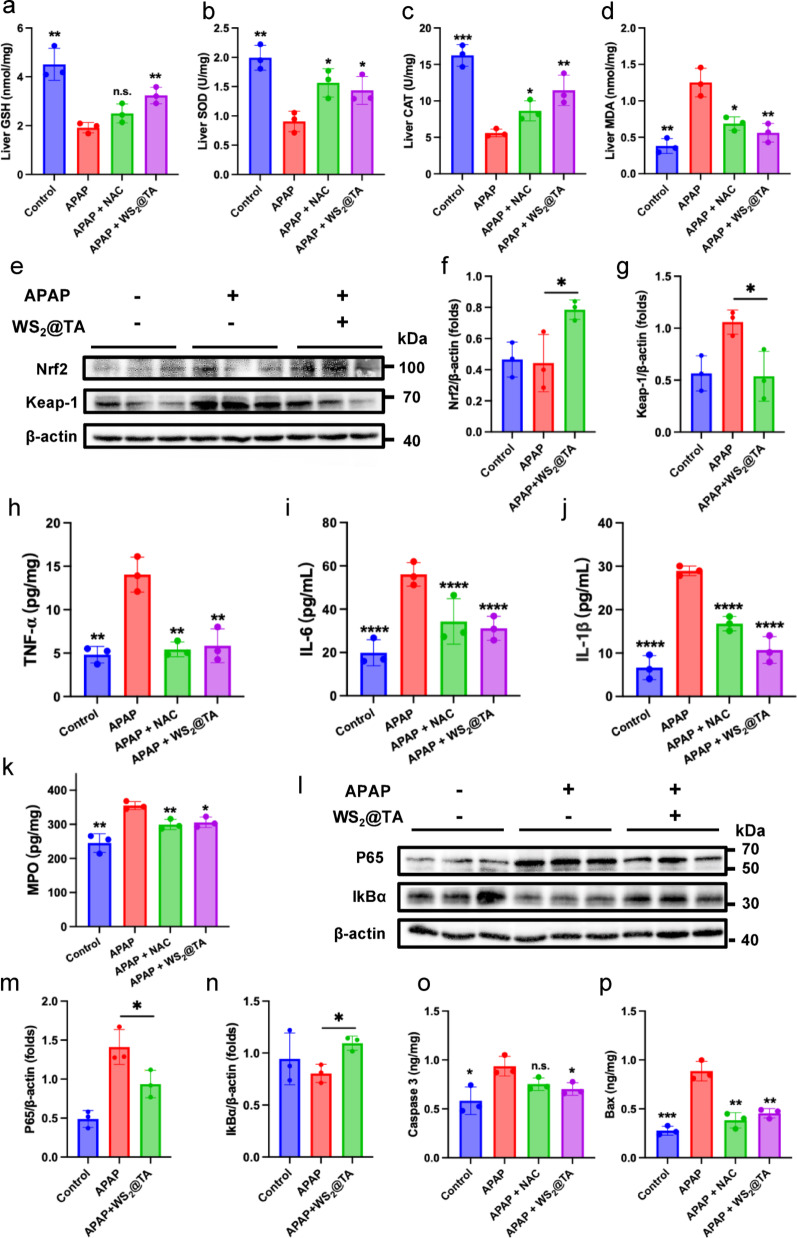
WS_2_@TA NSs activate Keap1-Nrf2 and inhibit the NF-κB signaling pathway to achieve synergistic inflammation inhibition. (**a**–**c**) Enzymatic activity levels of GSH, SOD and CAT in liver tissue (*n* = 3). (**d**) MDA levels of liver tissue (*n* = 3). (**e**-**g**) The expression levels of Nrf2 and Keap1 were assessed through WB. Gray values were quantitatively analyzed via ImageJ software. All data were normalized using β-actin as an internal reference. (**h**–**j**) Determination of liver tissue TNF-α, IL-6, and 1 L-1β through ELISA (*n* = 3). (**k**) Determination of liver tissue MPO activity by ELISA. (**l**-**n**) The expression levels of IκBα and p65 were assessed through WB and gray values analysis. All data were normalized using β-actin as an internal reference. (**o**) Caspase 3 and (**p**) Bax activity in homogenized liver tissues

GSH, SOD, and CAT are important antioxidant enzymes and antioxidants in the human body, which protect hepatocytes and promote liver repair by counteracting oxidative stress, scavenging free radicals, and maintaining intracellular redox homeostasis in liver injury. The decrease of these antioxidants is a key feature of oxidative stress caused by APAP. In the advanced-stage AILI mice model, treatment with WS_2_@TA NSs nanomedicines significantly elevated the content of GSH, SOD, and CAT in liver tissues (Fig. 6a-c). Notably, WS_2_@TA NSs also demonstrated a therapeutic effect comparable to that of NAC at lower doses, highlighting its potential application in counteracting APAP-induced oxidative damage. MDA is an important biomarker of oxidative stress, which not only reflects the stress-relieving efficacy of antioxidants but also provides insight into the degree of lipid peroxidation-induced damage in tissues. Compared with the control group, the APAP group demonstrated markedly elevated levels of MDA concentrations, suggesting that significant cellular damage due to lipid peroxidation transpired within the liver tissues. Conversely, the concentration of MDA was notably decreased in the WS_2_@TA NSs-treated group (Fig. 6d). In order to deeply investigate the molecular mechanism, the WB technique was employed to test the hypothesis. The experimental results showed that WS_2_@TA NSs significantly increased the expression level of Nrf2 and correspondingly decreased the expression of Keap1 (Fig. 6e-g). These findings confirmed that WS_2_@TA NSs significantly improved therapeutic efficacy by regulating the Nrf2-Keap1 signaling pathway. Intracellular RONS increase during liver injury process, leading to lipid peroxidation of cell membranes and disruption of cellular structure and function. This oxidative stress activates Kupffer cells (macrophages in the liver), which release pro-inflammatory factors such as TNF-α, IL-6, and IL-1β, further exacerbating the inflammatory response. To verify whether WS_2_@TA NSs could ameliorate liver injury by inhibiting inflammatory factors, the assay results showed, as expected, that the treatment of WS_2_@TA NSs significantly reduced the production of TNF-α, IL-6, and IL-1β pro-inflammatory factors in the livers of AILI mice (Fig. 6h-j). In addition, WS_2_@TA NSs effectively inhibited MPO activity, indicating that inflammatory cell infiltration was suppressed (Fig. 6k). The molecular mechanism was further elucidated by the WB technique. The APAP-induced upregulation of p65 and downregulation of IκBα expression were reversed after WS_2_@TA NSs treatment (Fig. 6l-n). These findings indicate that the protective effect of WS_2_@TA NSs against APAP-induced liver inflammation is significantly related to the suppression of the NF-κB signaling pathway. We also evaluated the WS_2_@TA NSs effects on the apoptotic pathway. The findings indicated a notable upregulation of Bax and Caspase-3 expression in mice subjected to APAP treatment, however, treatment with WS_2_@TA NSs counteracted these changes (Fig. 6o, p). These observations suggest that WS_2_@TA NSs contribute to cell survival and facilitate the reversal of AILI.

Recent studies have confirmed the existence of complex interaction between the Nrf2 and NF-κB pathways, which confirmed that Nrf2 deficiency enhances p65-mediated NF-κB signaling activity [38]. Additionally, we employed clobetasol propionate (CP) as an Nrf2 inhibitor for verification of causality with pathway perturbation [39]. Mice received PBS, WS_2_@TA NSs (intravenous injection, 2.5 mg/kg) or CP (intraperitoneal injection, 1 mg/kg) after 2 h of APAP intoxication. Mice were executed 24 h after APAP treatment and liver tissue sections were made and stained with TUNEL (green). TUNEL staining analysis revealed minimal TUNEL-positive cell expression in the liver tissue of control group (Fig. S18). Conversely, APAP stimulation induced extensive TUNEL-positive cell expression, which was reversed by WS_2_@TA NSs pretreatment. TUNEL assays revealed that the pre-injection of CP significantly diminished the therapeutic efficacy (protective effect against hepatocyte apoptosis induced by excessive APAP stimulation) of WS_2_@TA in AILI mice. This result directly demonstrates that the therapeutic effect of WS_2_@TA indeed depends on Nrf2 pathway activation. There is an interaction between the Nrf2 pathway and the NF-κB pathway, after Nrf2 inhibition, the NF-κB pathway inhibition benefits from WS_2_@TA NSs are abrogated. In summary, WS_2_@TA NSs is similar to other antioxidant nanomedicines that have been reported [19, 20], which can effectively suppressed oxidative stress induced by APAP overdose by indirectly reducing RONS levels, relieving Keap1 inhibition of Nrf2, and activating antioxidant signaling pathways.

## Conclusions

In summary, we have established a liquid-phase stripping methodology for the synthesis of WS_2_@TA NSs with a high specific surface area and abundant phenolic moieties in a 2D structure. Subsequently, we investigated the potential application of WS_2_@TA NSs as highly effective anti-inflammatory nano-agents for the targeted enrichment therapy of AILI lesions. Due to the valence shift from W^4+^ to W^6+^ and the synergistic oxidative reaction of S^2−^ on their surfaces, along with the negative electronegativity brought by TA modification, the obtained WS_2_@TA NSs possess highly efficient RONS scavenging ability and excellent targeting ability to the inflamed colon, respectively. What’s more, WS_2_@TA NSs can be efficiently excreted through the kidney and liver-intestinal system, which ensures their safety for the treatment of AILI. Excitingly, in the advanced-stage AILI mice model, WS_2_@TA NSs could activate Keap1-Nrf2 and inhibit the NF-κB signaling pathway, thereby achieving synergistic inflammation inhibition and alleviating the advanced-stage AILI mice model. Moreover, the survival rate of mice treated with WS_2_@TA was also superior to that of mice treated with NAC among the APAP (600 mg/kg) lethal dose mice model. This study offers valuable insights into the potential applications of tungsten-based nanomedicines for anti-inflammatory purposes, and WS_2_@TA NSs is a novel potential candidate for late-stage AILI with an excellent biosafety profile.

## Supplementary Information

Supplementary Material 1.

## Data Availability

No datasets were generated or analysed during the current study.
