# Supplementary material for: Tungsten disulfide atomic crystals with RONS scavenging and liver targeting capabilities for acetaminophen-induced acute liver injury therapy
Source: J Nanobiotechnology. 2025 Oct 11;23:665. doi: 10.1186/s12951-025-03771-7 (PMC12514798; doi:10.1186/s12951-025-03771-7)
Supplement: Supplementary file 1 — Supplementary Material 1. [file 12951_2025_3771_MOESM1_ESM.docx]

**Supporting Information**

**Tungsten disulfide atomic crystals with RONS scavenging and liver targeting capabilities for acetaminophen-induced acute** **liver injury therapy**

Ziwen Xiao ^a,#^, Yu Liu ^a,#^, Zhenchao Tao ^b,#^, Yu Zhang ^a^, Qian Chen ^a^, Zhaohua Miao ^a^, Zhengbao Zha ^a^, Yan Ma ^c*^, Hua Wang ^d*^, Deyan Gong ^a*^

^a^ School of Food and Biological Engineering, Hefei University of Technology, Hefei 230009, China

^b^ Department of Radiation Oncology, Anhui Provincial Cancer Hospital, Hefei 230031, China

^c^ School of Biomedical Engineering, Anhui Medical University, Hefei, 230022 China

^d^ Department of Oncology, The First Affiliated Hospital of Anhui Medical University, Hefei 230032, China

^#^These authors contributed equally

^*^Corresponding authors: [may@ahmu.edu.cn](mailto:may@ahmu.edu.cn) (Yan Ma); [wanghua@ahmu.edu.cn](mailto:wanghua@ahmu.edu.cn) (Hua Wang); [gongdy@hfut.edu.cn](mailto:gongdy@hfut.edu.cn) (Deyan Gong)


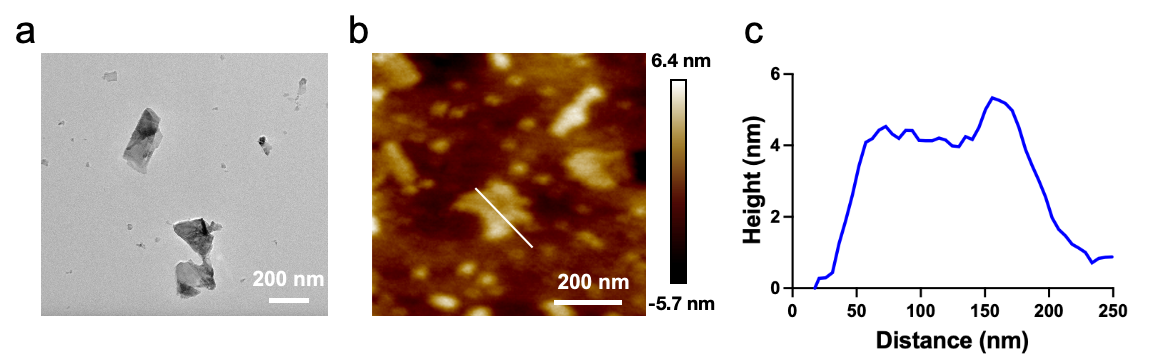


**Figure S1.** (a) TEM image of WS_2_ NSs. (b) AFM image and (c) the corresponding thickness profiles of WS_2_ NSs.


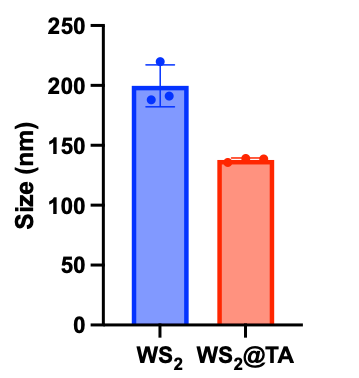


**Figure S2.** Hydrated particle size of WS_2_ NSs and WS_2_@TA NSs.


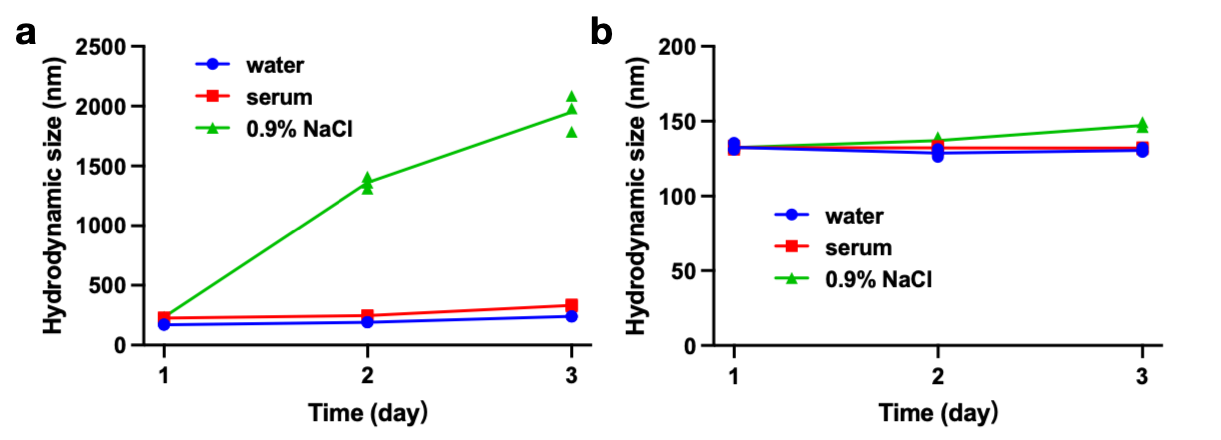


**Figure S3.** (a) The variation of hydration particle size of WS_2_ NSs in different media. (b)The variation of hydration particle size of WS_2_@TA NSs in different media.


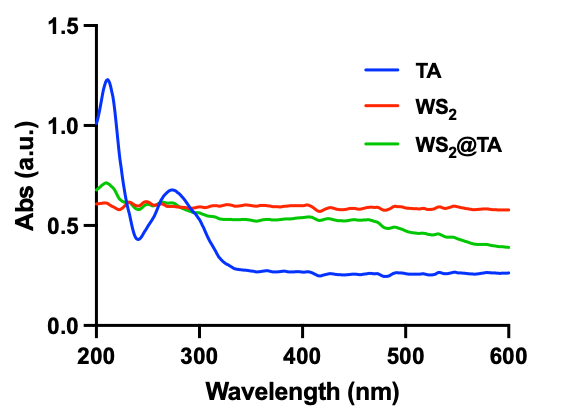


**Figure S4.** UV−vis spectra of WS_2_@TA NSs, WS_2_ NSs, and TA.


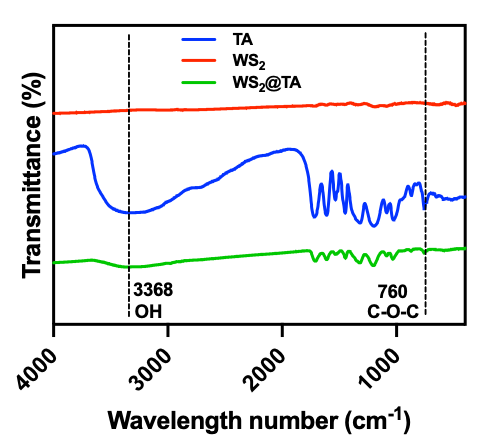


**Figure S5.** Fourier transform infrared (FTIR) spectra of WS_2_@TA NSs, WS_2_ NSs, and TA.


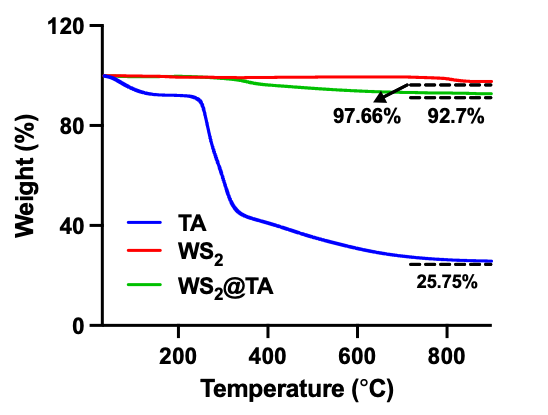


**Figure S6.** Thermogravimetric (TG) analysis of WS_2_@TA NSs, WS_2_ NSs, and TA.


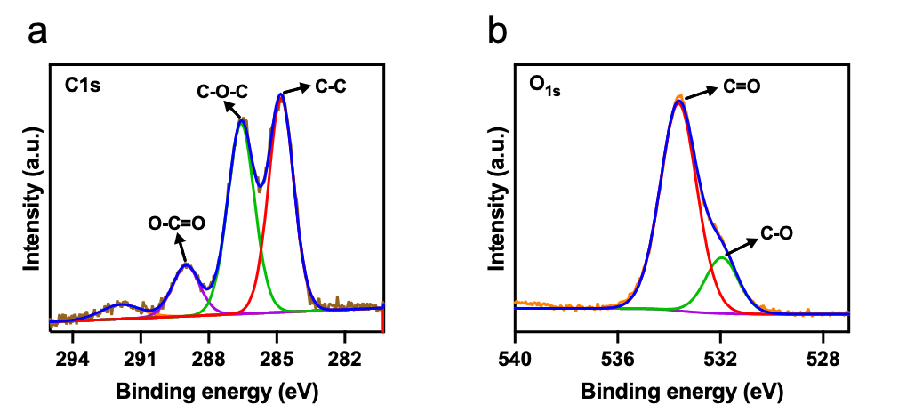


**Figure S7.** (a) Core XPS spectra of C element. (b) Core XPS spectra of O element.


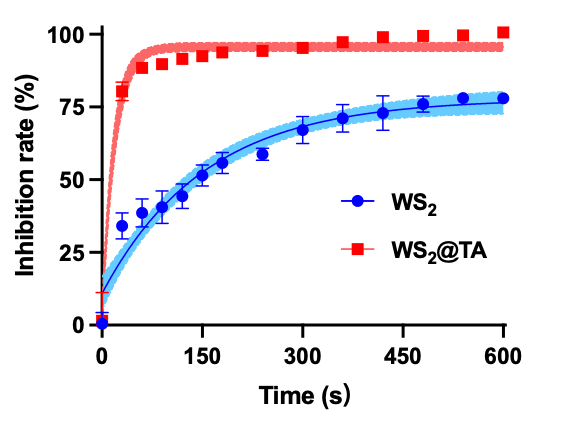


**Figure S8.** The clearance kinetics curve of DPPH.


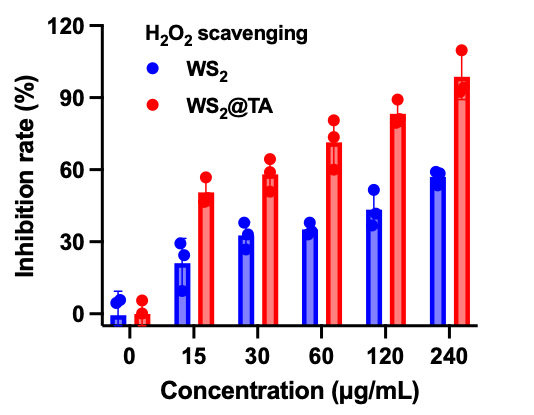


**Figure S9.** H_2_O_2_ scavenging efficiencies.


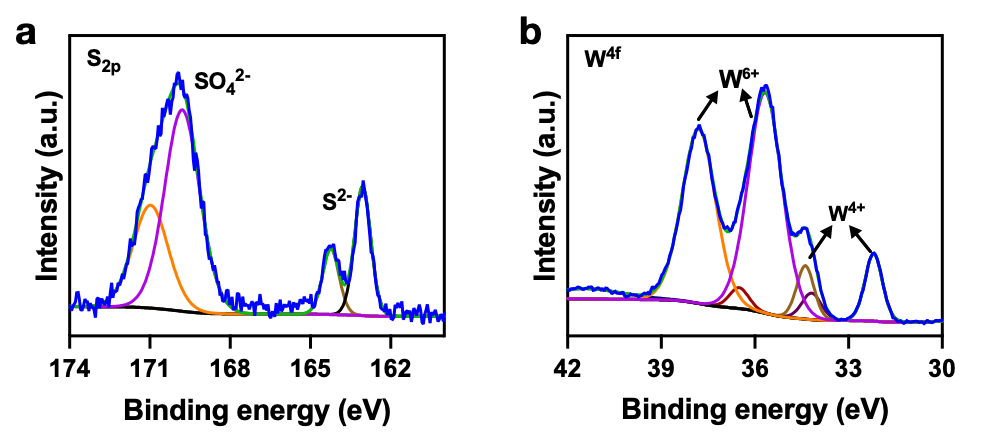


**Figure S10**. (a) Core XPS spectra of S element and (b) W element in WS_2_@TA after H_2_O_2_ treatment.


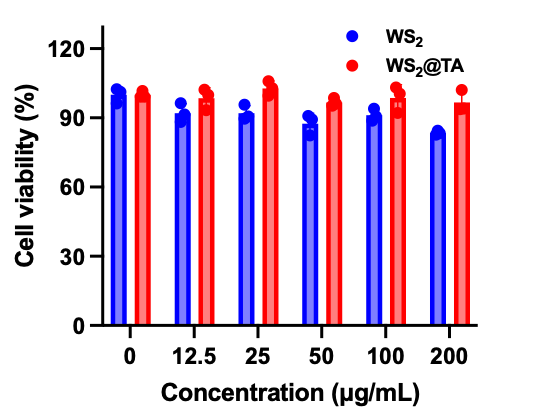


**Figure S11**. Cell viability of Raw 264.7 cells treated with WS_2_ NSs or WS_2_@TA NSs of different concentrations (n = 3).


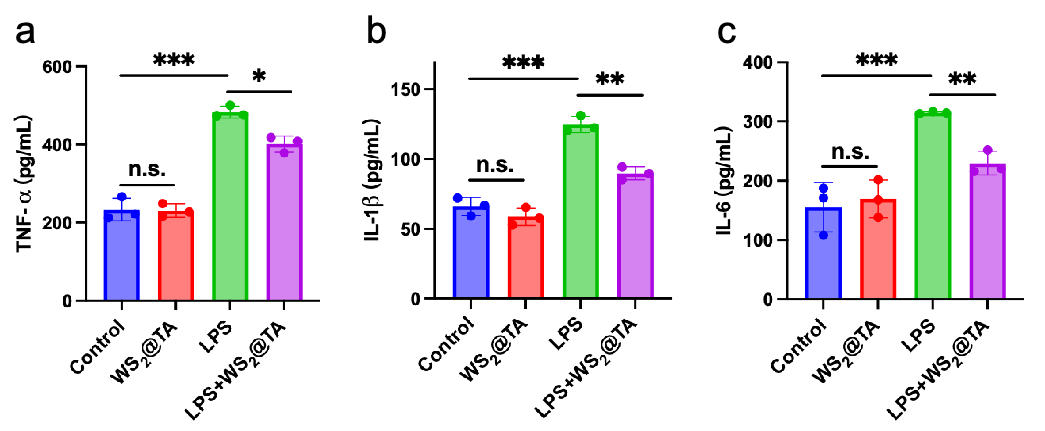


**Figure S12.** (a-c) Downregulation of LPS-induced proinflammatory cytokine (TNF-α, IL-1β, and IL-6) levels.


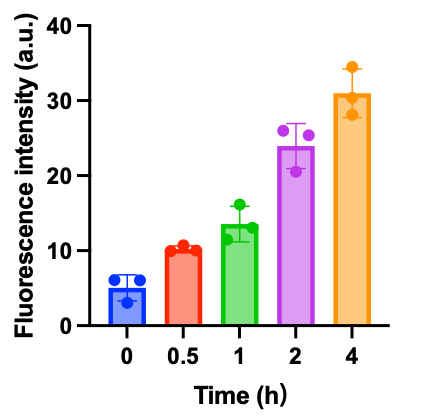


**Figure S13.** Quantitative analysis of the fluorescence intensity of intracellular Cy5.5-labeled WS_2_@TA NSs (n = 3).


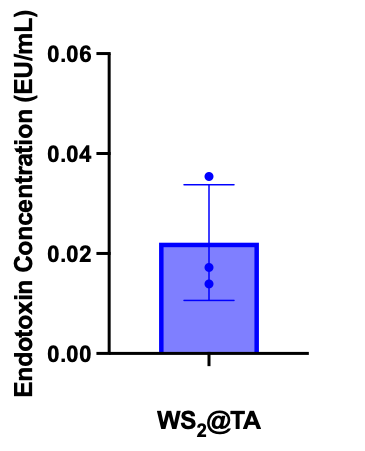


**Figure S14.** Endotoxin activity in 0.5mg/mL WS_2_@TA solution.


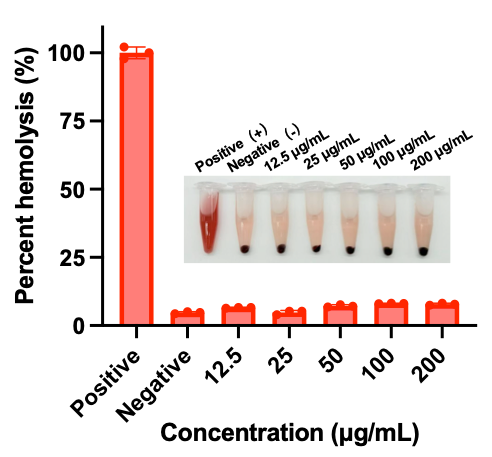


**Figure S15.** Hemolysis test of WS_2_@TA NSs.


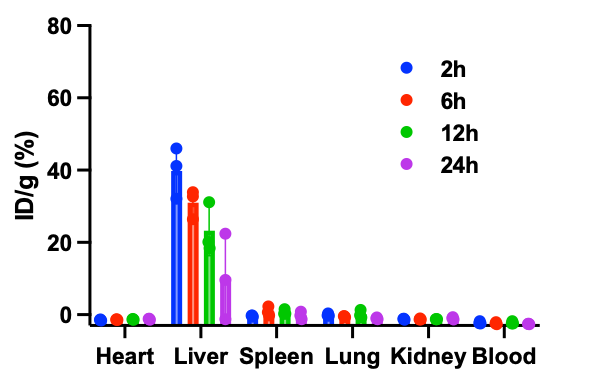


**Figure S16.** Distribution of W element in organs of AILI mice after intravenous injection of WS_2_ NSs (n=3).


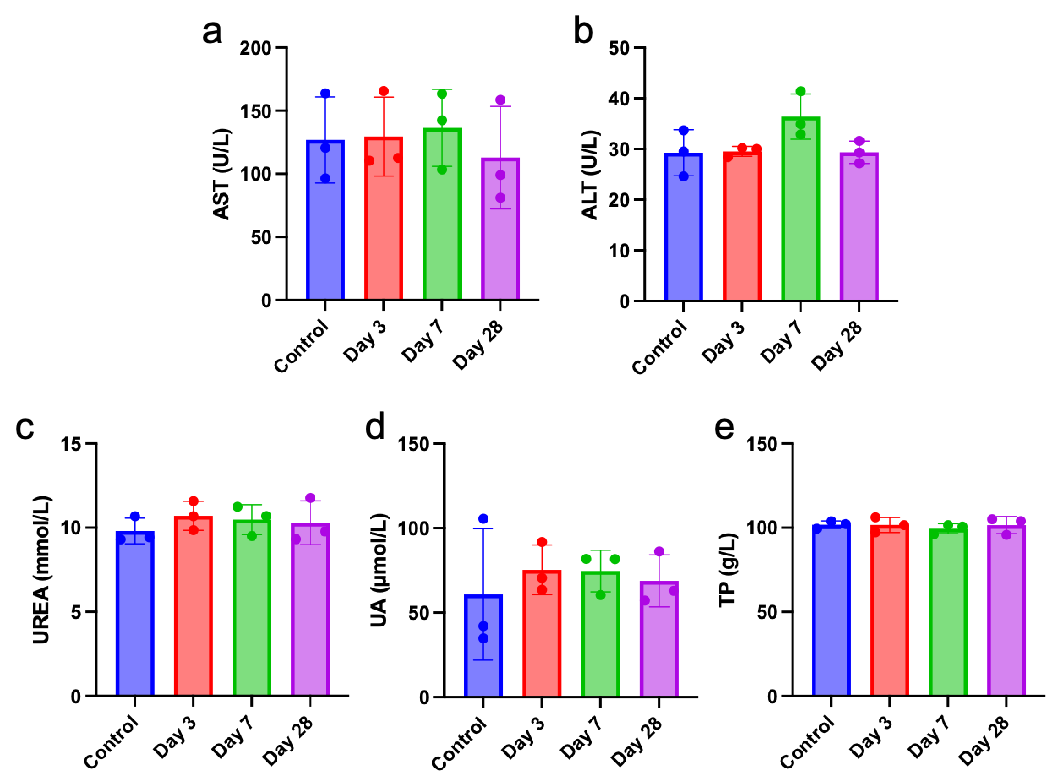


**Figure S17.** Blood biochemistry index tests. (a) Serum levels of ALT, (b) AST, (c) UREA, (d) UA and (e) TP.


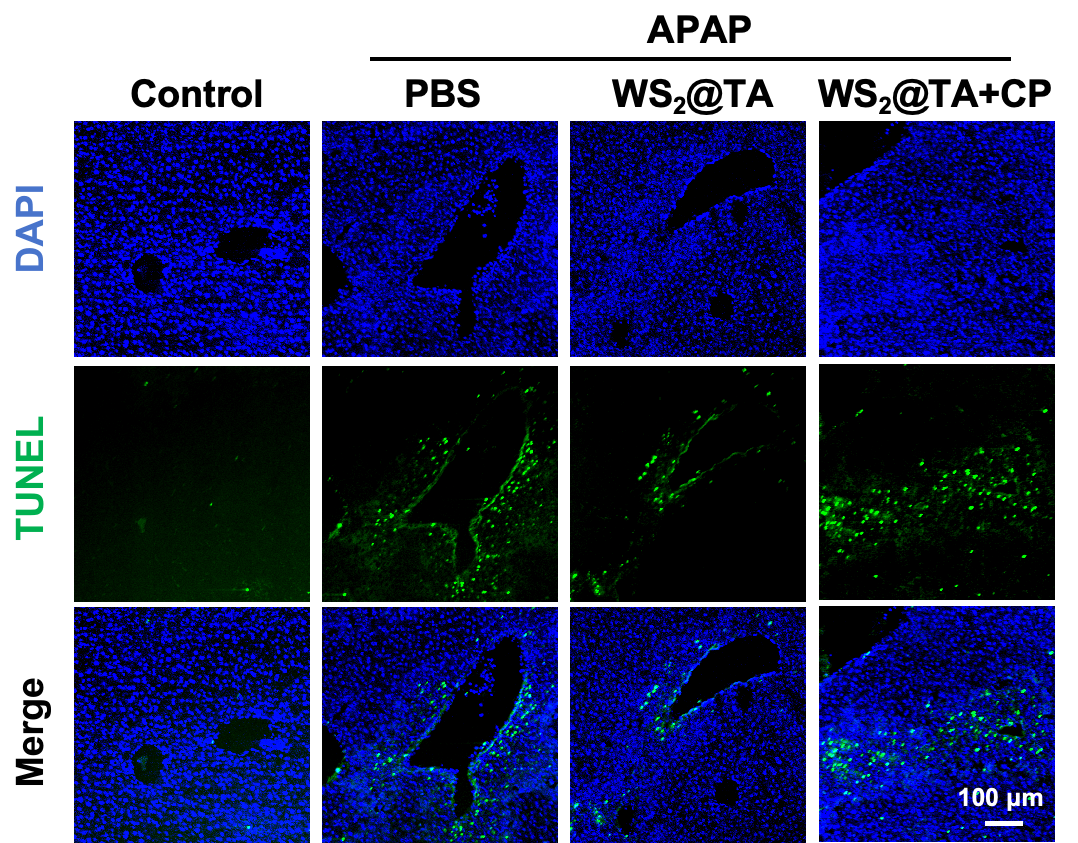


**Figure S18.** Representative images of TUNEL fluorescence imaging of liver tissue sections.
